# The structure of a novel antibody against the spike protein inhibits Middle East respiratory syndrome coronavirus infections

**DOI:** 10.1038/s41598-022-05318-4

**Published:** 2022-01-24

**Authors:** Tae-Ho Jang, Woo-Jung Park, Hansaem Lee, Hye-Min Woo, So-young Lee, Kyung-Chang Kim, Sung Soon Kim, Eunmi Hong, Jaeyoung Song, Joo-Yeon Lee

**Affiliations:** 1 New Drug Development Center, Daegu-Gyeongbuk Medical Innovation Foundation, Daegu, Republic of Korea; 2 Division of Emerging Virus and Vector Research, Center for Emerging Virus Research, Korea National Institute of Health, Korea Disease Control and Prevention Agency, Cheongju-si, Republic of Korea; 3 Center for Emerging Virus Research, Korea National Institute of Health, Korea Disease Control and Prevention Agency, Cheongju-si, Republic of Korea; 4 Center for Vaccine Research, Korea National Institute of Health, Korea Disease Control and Prevention Agency, Cheongju-si, Republic of Korea

**Keywords:** Immunology, Molecular biology, Structural biology

## Abstract

Middle East respiratory syndrome coronavirus (MERS-CoV) is a zoonotic virus, responsible for outbreaks of a severe respiratory illness in humans with a fatality rate of 30%. Currently, there are no vaccines or United States food and drug administration (FDA)-approved therapeutics for humans. The spike protein displayed on the surface of MERS-CoV functions in the attachment and fusion of virions to host cellular membranes and is the target of the host antibody response. Here, we provide a molecular method for neutralizing MERS-CoV through potent antibody-mediated targeting of the receptor-binding subdomain (RBD) of the spike protein. The structural characterization of the neutralizing antibody (KNIH90-F1) complexed with RBD using X-ray crystallography revealed three critical epitopes (D509, R511, and E513) in the RBD region of the spike protein. Further investigation of MERS-CoV mutants that escaped neutralization by the antibody supported the identification of these epitopes in the RBD region. The neutralizing activity of this antibody is solely provided by these specific molecular structures. This work should contribute to the development of vaccines or therapeutic antibodies for MERS-CoV.

## Introduction

Middle East respiratory syndrome coronavirus (MERS-CoV) is a zoonotic virus belonging to the betacoronaviridae family and can infect bats, dromedary camels, and humans^1–3^. MERS-CoV, which causes severe pulmonary illness and renal failure, has a global fatality rate of 30%^4^. Unlike other human coronaviruses, MERS-CoV uses human dipeptidase 4 (hDPP4 also known as CD26) as the main host receptor^5^, and has a wide tissue tropism in the human body, infecting the lung, liver, and kidneys^6,7^. Since the initial outbreak of MERS-CoV in Saudi Arabia in 2012, the virus has spread to 27 countries, resulting in 2494 laboratory-confirmed cases and 858 deaths (January 2020, WHO)^8^. In addition, the biggest MERS-CoV outbreak outside of the Middle East occurred in South Korea in 2015^9^. Hundreds of MERS-CoV outbreaks have occurred continuously in the Middle East, especially in Saudi Arabia, suggesting that future occurrences of MERS-CoV are likely. Furthermore, since 2015, the world health organization (WHO) R&D blueprint has declared MERS-CoV to be one of the highest priority infectious diseases for vaccine and therapeutics research and development in the prevention of threats global public health^10^. There are no clinically approved vaccines or therapeutic agents so far developed for MERS-CoV.

At the initial infection stage, MERS-CoV uses receptors on its spike (S) protein to attach and fuse its envelope with the cellular membrane and deliver its genome into the host cell. The S1 region (from amino acids 14–756) of the S protein acts as an attachment and, like all class I fusion proteins, S2 (from amino acids 757–1351) is a key a fusion protein facilitating the action of the entry machinery^11^. The S protein is synthesized as a premature precursor and cleaved by several proteases, such as type II transmembrane serine proteases and furin, to form S1 and S2 on the membrane surface. These conformational changes are involved in the fusion step^12–14^ and lead to S2 triggering S2 hydrophobic fusion to the cellular membrane^11,15^. The S protein is a type I homotrimeric transmembrane protein with an S1ectodomain, an S2 stalk, and a C-terminal transmembrane region^15^. The receptor-binding domain (amino acids E367 to Y606) of the S1 domain is responsible for binding to the hDPP4 receptor^16^, and the N-terminal region (amino acids 14–366) is known to bind to the cellular receptor alpha 2,3-linked-sialic acid^17^.

S protein is the main target of neutralizing antibodies (nAb) in the protective immune response to MERS-CoV, and various human monoclonal antibodies have been developed as MERS-CoV therapeutics^18–30^. Of these, KNIH90-F1 was previously isolated from B cells of a Korean convalescent MERS patient and was shown to neutralize MERS-CoV in vitro by interfering with the RBD of the S protein and hDPP4 interaction^30^. Moreover, KNIH90-F1 protected hDPP4-expressing transgenic mice from MERS-CoV lethal challenges with high potency.

To identify the critical epitopes of KNIH90-F1 in detail, we performed X-ray crystallography analysis of the MERS-CoV RBD and KNIH90-F1 antigen-binding fragment (Fab) complex. The structural data and the results of analyzing MERS-CoV mutants that escaped KNIH90-F1 antibody treatment demonstrated that KNIH90-F1 binds directly to RBD and inhibits the attachment of MERS-CoV to the hDPP4 receptors. These results allowed us to define the neutralizing epitope of KNIH90-F1 and pave the way for the practical use of KNIH90-F1 as a therapeutic or prophylactic agent to treat MERS-CoV-infected individuals.

## Results

### Structural elucidation of MERS-CoV RBD complexed with KNIH90-F1 Fab

**Figure 1:**
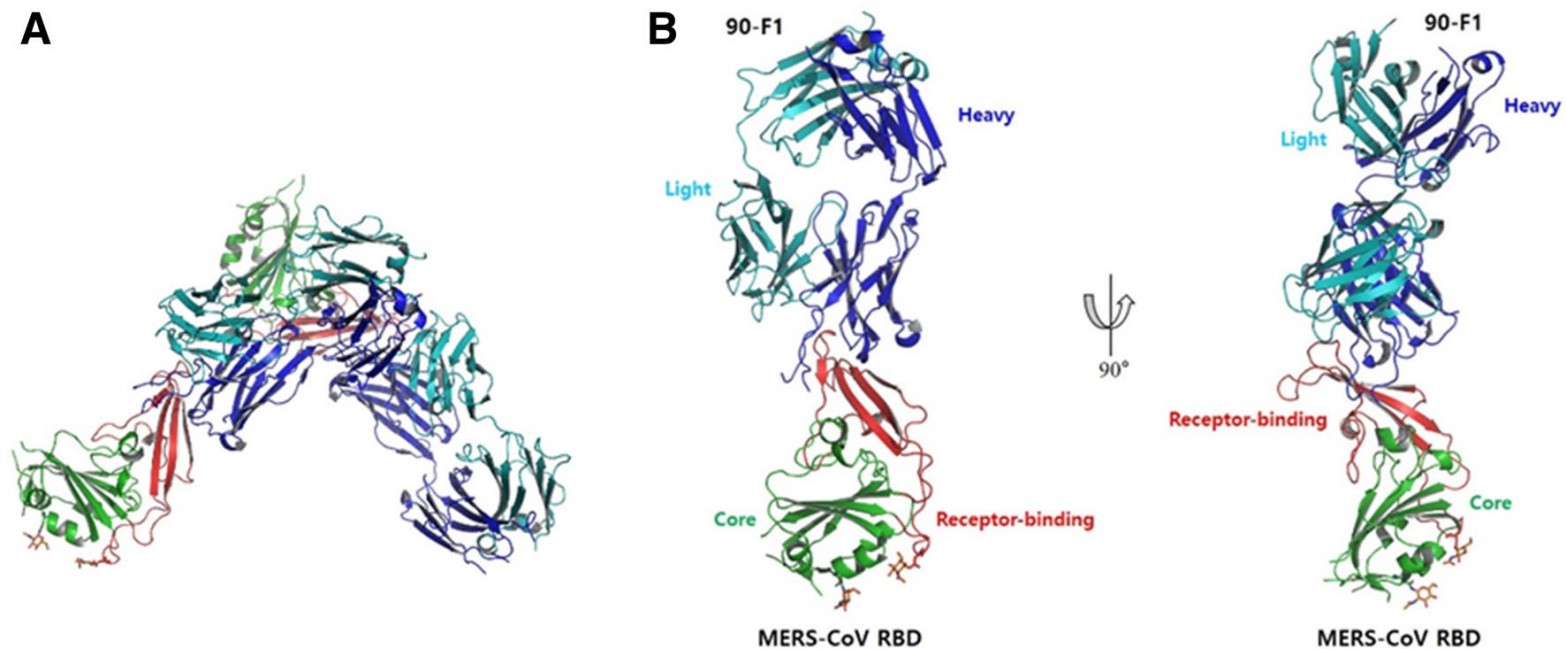
Crystal structure of MERS-CoV RBD complexed with KNIH90-F1 Fab. (**A**) Dimeric biological assembly of MERS-CoV RBD. Asymmetric unit containing a single RBD bound to a single KNIH90-F1 Fab to form a dimeric assembly. (**B**) The structure of MERS-CoV RBD complexed with KNIH90-F1 Fab. The complex comprises core/receptor-binding subdomains in the RBD and light/heavy chains of KNIH90-F1 Fab.

**Figure 2:**
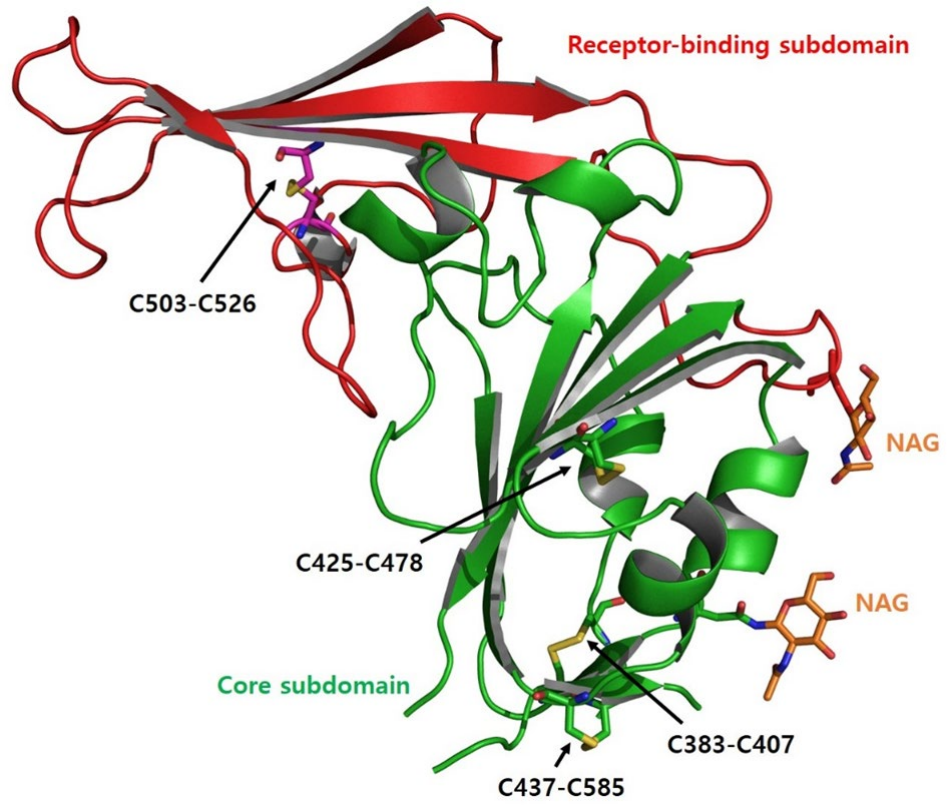
The structure of MERS-CoV RBD. Eight Cys residues in RBD form disulfide bonds in the core subdomain (Cys383-Cys407, Cys425-Cys478, Cys437-Cys585) and receptor-binding domain (Cys502-Cys526), and the disulfide bonds stabilize the overall structure of RBD. Two NAGs form covalent bonds with Asn410 and Asn487 side chains.

To gain structural insight into how KNIH90-F1 Fab neutralizes MERS-CoV, we solved the structure of MERS-CoV RBD (amino acids 367 to 588) complexed with the KNIH90-F1 Fab at 2.05 Å resolution by molecular replacement using a searching model (PDB ID: 4ZS6). The collected data were refined and converged to final *R*_work_ = 0.17% and *R*_free_ = 0.22% using Coot and Phenix. All figures were generated by Pymol (version 2.3.2)^31^. Structural analysis revealed an asymmetric unit containing a single RBD bound to a single KNIH90-F1 Fab to form a dimeric biological assembly (Fig. 1A). The complex comprised residues Val381-Lys587 of RBD, residues Gln1-Lys232 of the KNIH90-F1 heavy chain, and residues Glu1-Gly210 of the KNIH90-F1 light chain, with a chain break at residues 579–580 in RBD and residues 107–108, 145–146, and 207–208 in the heavy chain due to the lack of electron density as a result of the flexibility of the regions (Fig. 1B). In addition, two molecules of N-acetyl-D-glucosamine (NAG) formed separate side chains through covalent bonds with Asn410 and Asn487. MERS-CoV RBD has been reported to consist of a core subdomain containing five-stranded anti-parallel sheets and five short helices, and a receptor-binding subdomain of four-stranded anti-parallel sheets and one short helix^27,32^. In our study, the structure of the MERS-CoV RBD complexed with the KNIH90-F1 Fab fragment consisted of a core RBD subdomain comprising five-stranded anti-parallel sheets, five short helices, and two short-stranded parallel sheets at the C- and N-terminals. The receptor-binding subdomain of RBD included four-stranded anti-parallel sheets and one short helix. Eight Cys residues in RBD formed disulfide bonds in the core subdomain (Cys383-Cys407, Cys425-Cys478, Cys437-Cys585) and receptor-binding domain (Cys502-Cys526), resulting in RBD having an overall globular structure, and no free Cys residues were found (Fig. 2). Three disulfide bonds in the core subdomain stabilized the core domain structure, and a disulfide bond in the receptor-binding subdomain increased the rigidity of a long flexible loop region clutched to an adjacent stranded sheet, which stabilized the overall structure of the receptor-binding subdomain.

### Biding site of MERS-CoV RBD to KNIH90-F1 Fab

**Figure 3:**
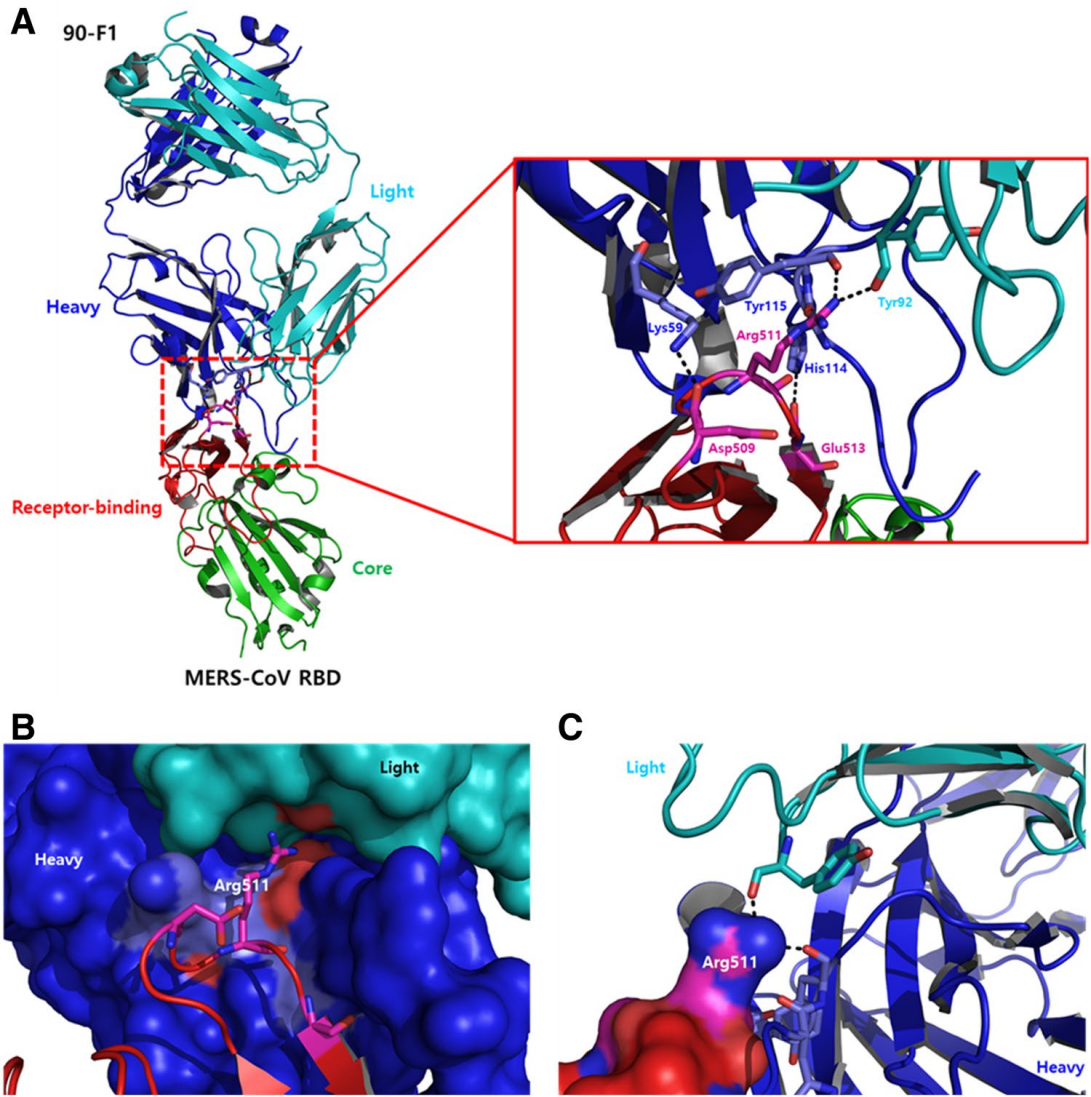
Epitope region of MERS-CoV RBD in a complex with KNIH90-F1 Fab. (**A**) Asp509, Arg511, and Glu513 in RBD are key residues binding residues in the heavy and light chains of KNIH90-F1 Fab. (**B**, **C**) Arg511 plays a critical role in enhancing the interaction between RBD and KNIH90-F1 Fab.

**Figure 4:**
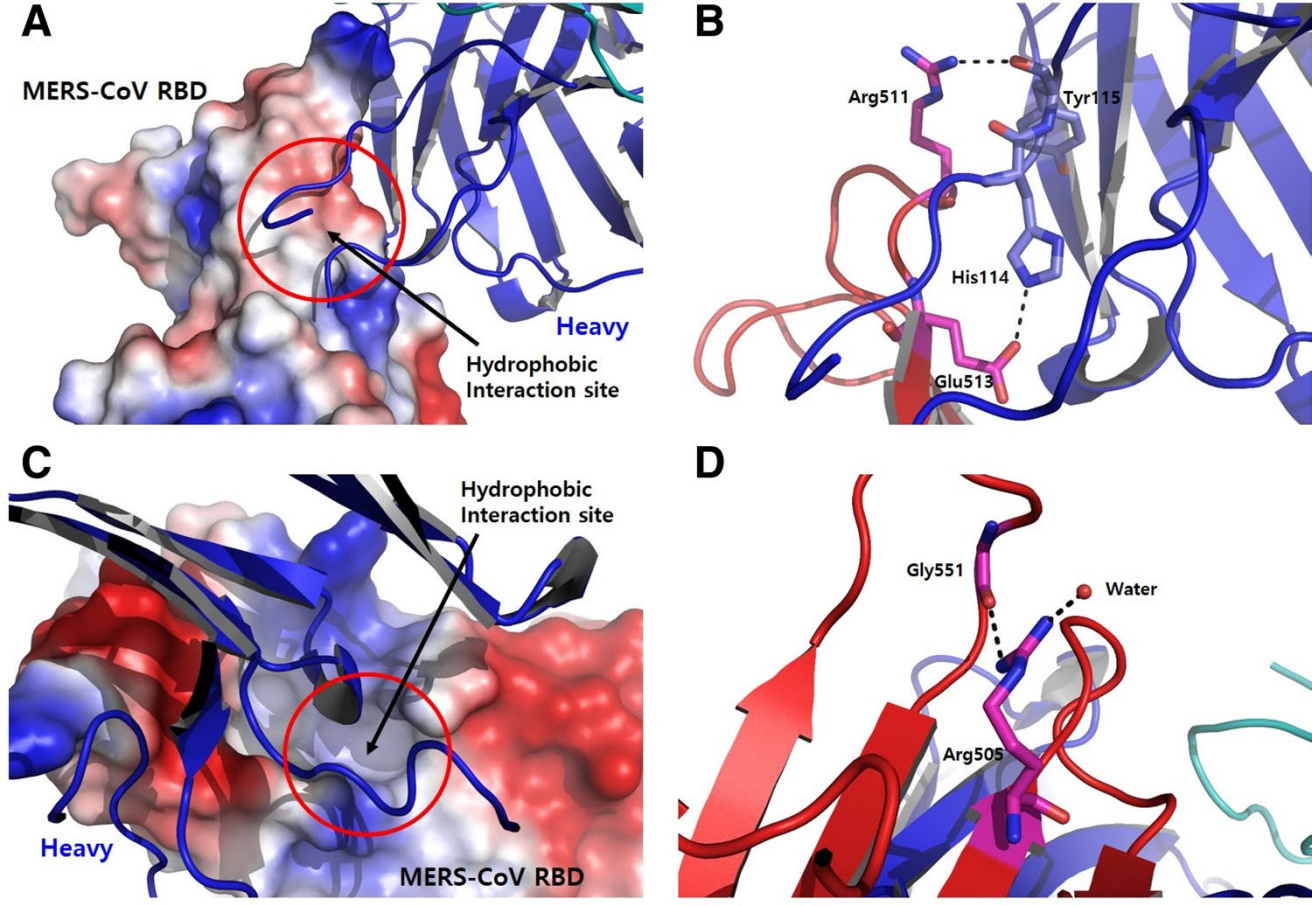
The hydrophobic interaction sites of MERS-CoV RBD complexed with KNIH90-F1 Fab. (**A**) The hydrophobic residues in the heavy chain of KNIH90-F1 Fab make contact with the hydrophobic region in the receptor-binding subdomain of RBD. (**B**) The hydrophobic interaction in (**A**) is reinforced by the interactions between Arg511 in RBD and Tyr115 in the heavy chain and between Glu513 in RBD and His114 in the heavy chain. (**C**) An additional hydrophobic interaction site between the receptor-binding subdomain of RBD and KNIH90-F1 Fab. (**D**) A key interaction between the Arg505 side chain, Gly551, and a water molecule induces the hydrophobic groove in (**C**).

Structural analysis of RBD in the complex with KNIH90-F1 Fab confirmed that MERS-CoV RBD clearly binds to KNIH90-F1 Fab. Both the heavy and light chains of KNIH90-F1 Fab participated in interactions with the key residues of the receptor-binding subdomain of RBD. The key residues Asp509, Arg511, and Glu513 in RBD bound to adjacent residues in the heavy and light chains of KNIH90-F1 Fab. The carbonyl group of the Asp509 main chain and Glu513 side chain in RBD respectively interacted with Lys59 and His60 in the heavy chain at 2.8 Å by hydrogen bonding. Additionally, an amine of the Arg511 side chain interacted with a carbonyl group of Tyr115 in the heavy chain and Tyr92 in the light chain at 2.7 Å and 2.8 Å, respectively, by hydrogen bonding (Fig. 3A). In particular, the positive protruding region in RBD induced by the Arg511 side chain mainly played a role in the hinge buried by both the heavy and light chains, enhancing the overall interaction between RBD and KNIH90-F1 Fab (Fig. 3B,C). The hydrophobic residues in the heavy chain of KNIH90-F1 Fab, i.e., Ser105, Tyr106, Gly109, Ser110, Tyr111, Tyr112, and Thr113, made contact with the hydrophobic groove in the receptor-binding subdomain of RBD, which contained Thr512, Val514, Pro515, and Leu517. The hydrophobic interaction was reinforced by key interactions between Arg511 in RBD and Tyr115 in the heavy chain and between Glu513 in RBD and His114 in the heavy chain (Fig. 4A,B). Further hydrophobic residues in the receptor-binding subdomain of RBD, Leu506 and Leu507, formed a hydrophobic groove. Interestingly, the Arg505 side chain in RBD moved toward the opposite side of the binding region with the heavy chain of KNIH90-F1 Fab. One amine group of the Arg505 side chain interacted with a proximal carbonyl of Gly551 (2.9 Å) by hydrogen bonding, and the other amine group of the Arg505 side chain interacted with an adjacent water molecule at 2.3 Å by hydrogen bonding (Fig. 4C,D). Also, T512 and L506 of RBD residues are in hydrophobic region of RBD and participated in hydrophobic interaction with KNIH90-F1 in the complex (Table 1).

**Table 1:** Data collection and refinement statistics.

Data collection	
Wavelength (Å)	0.97960
Spece group	P1 21 1
Cell dimensions	
a, b, c (Å)	71.59, 146.819, 81.622
α, β, γ (°)	90, 96.732, 90
Total reflections	105,587 (9884)
Unique reflections	103,522 (9762)
Completeness (%)	98.95 (93.74)
Mean I/sigma(I)	14.97 (3.02)
R_merge_	0.101 (0.312)
CC1/2	0.0992 (0.896)
CC*	0.0998 (0.972)
Resolution range (Å)	44.95–2.05 (2.123–2.05)
R_work_	0.1764 (0.2244)
R_free_	0.2228 (0.2698)
CC(work)	0.956 (0.899)
CC(free)	0.938 (0.826)
Number of non-hydrogen atoms	10,840
Macromolecules	9698
Ligands	172
Solvent	970
RMS (bonds)	0.007
RMS (angles)	0.91
Ramachandran favored (%)	97.22
Ramachandran allowed (%)	2.78
Ramachandran outliers (%)	0.00
Rotamer outliers (%)	0.00
Clashscore	2.52
Average B-factor	38.45
Macromolecules	37.64
Ligands	61.24
Solvent	42.43

*Statistics for the highest-resolution shell are shown in parentheses.

### Generation and characterization of antibody escape mutants

To indirectly confirm the identity of those residues critical to RBD-region binding to KNIH90-F1, we investigated the mutation of spike protein genes in viruses that escaped the nAb under the selective pressure of KNIH90-F1. After four consecutive passages in the presence of KNIH90-F1, we observed a cytopathic effect similar to that of the control virus on the cells from 2 days post-infection, indicating the sufficient generation of nAb escape mutants. Viruses were harvested from each passage and isolated by plaque purification. The amino acid sequences of the RBD regions of the isolated viruses were analyzed. Unexpectedly, only one of the six purified isolates had a mutation at a single amino acid (R511S) of RBD after one passage with nAb. However, at the final passage, three uniquely mutated viruses were isolated with various amino acid substitutions at the RBD region and were designated 1EM, 4EM, and 6EM. The 1EM virus had L506F, T512I, and E513A substitutions; the 4EM virus had L506F, R511L, and E513A substitutions; and the 6EM virus had R511S and T512A substitutions at the RBD region (Table 2). However, substitutions of consensus amino acid sequences of the RBD region were not observed in the control viruses passaged without nAb.

**Table 2:** Amino acid sequence of purified isolates during consensus passage in presence of nAb KNIH90-F1.

No. of passage	Mutated virus	Amino acid mutation on RBD region	No. of mutant/no. of plaque
1	–	R511S	1/6
		No mutation	5/6
2	–	L506F/E513A	3/6
		L506F/R511S/E513A	3/6
3	–	L506F/E513A	4/8
		L506F/R511L/E513A	1/8
		L506F/R511S/E513A	2/8
		R511S/T512A	1/8
4	1EM	L506F/T512I/E513A	1/8
	4EM	L506F/R511L/E513A	3/8
	6EM	R511S/T512A	4/8

**Figure 5:**
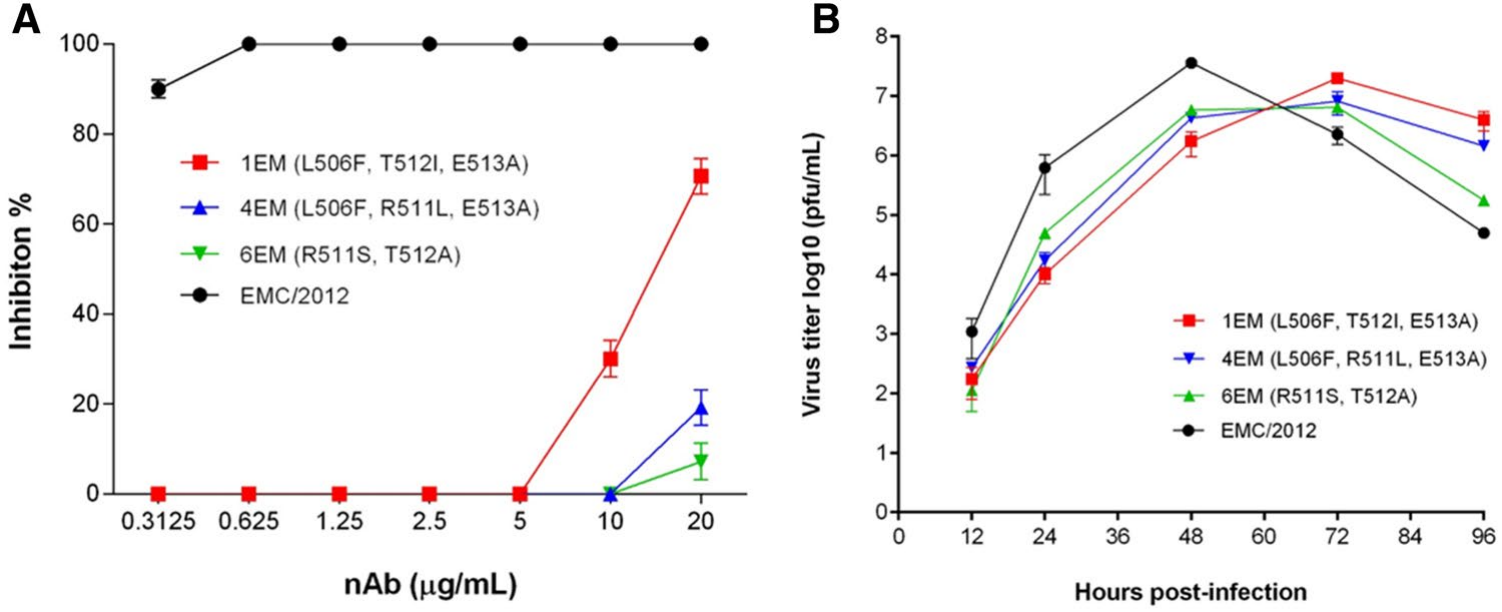
Characterization of MERS-CoV RBD variants generated by nAb KNIH90-F1 treatment. (**A**) Neutralization of three RBD mutants by the KNIH90-F1 neutralizing antibody was determined by plaque reduction neutralization test assay; 20 μg/ml of antibody was serially diluted twofold in PBS. (**B**) Growth kinetics of three RBD mutants. All viruses infected Vero cells at a multiplicity of infection of 0.01. Viral titers of cell culture supernatants were determined by plaque assay. EMC/2012 strain cultured without nAb was used as a control virus.

To further examine the escape of the three mutants from nAb KNIH90-F1 neutralization, we performed a PRNT assay. All RBD-mutation viruses completely escaped neutralization by high concentrations (5 μg/ml) of nAb, while infection by the wildtype virus was inhibited by the same concentration of KNIH90-F1. We observed a difference in the nAb-escape ability of the 1EM and 4EM viruses. The 1EM virus, with mutated L506F, T512I, and E513A, showed 70% inhibition with 20 μg/ml of nAb, whereas the 4EM virus, with a mutated R511L instead of T512I, was less inhibited at the same concentration of KNIH90-F1. The 6EM virus had mutations at both positions (R511S, T512A) and showed a similar degree of inhibition as the 4EM virus (Fig. 5A). This result indicates that not all the escaped mutants generated under the selective pressure of KNIH90-F1 were neutralized by the nAb. Moreover, position 511 of RBD appeared to have a critical role in the binding of KNIH90-F1 to the MERS-CoV RBD region.

Next, we determined whether the various mutations of these escape mutants affected their viral fitness in the cell culture system. All KNIH90-F1 escaped mutants achieved low titers compared with the EMC/2012 (wildtype) strain in the early stages. Moreover, while the EMC/2012 strain titer peaked at 48 h post-infection, all escape mutant titers peaked at 72 h post-infection, indicating that all the mutations resulted in impaired viral fitness according to kinetic and peak titers in Vero cells (Fig. 5B).

## Discussion

MERS-CoV continues to cause sporadic outbreaks in Middle Eastern countries, particularly Saudi Arabia, highlighting the urgent need for the development of both vaccines and therapeutics^10^. To combat the virus, several anti-viral strategies have been studied, utilizing synthetic antiviral agents to target proteases^33–35^; RNA-dependent RNA polymerase, such as Remdesivir^31,36^; or agents targeting the spike protein to inhibit viral entry. In particular, in vivo protection animal studies^18,19,24,25,29,32,37,38^ and clinical studies^39,40^, using therapeutic or polyclonal antibodies, have led to the development of neutralizing antibodies against MERS-CoV. We previously generated six human monoclonal antibodies that effectively neutralized MERS-CoV^30^. KNIH90-F1 is the most potent of these nAbs, with an IC_50_ of 6 ng/ml for live MERS-CoV, and it binds to the RBD of the spike protein. However, there was no direct evidence for the epitopes of this antibody. Several epitope sites (D510, 538GDY540, and W553) were characterized at the computational level in previous work.

In this study, we performed epitope mapping of the KNIH90-F1 antibody region that interacts with the RBD of the MERS-CoV spike region using X-ray crystallography and an escape mutant approach. Then, we identified the effects of the escape mutant characteristics on viral fitness. As previously described, the MERS-CoV RBD region contained key residues related to neutralizing epitopes, including L506, D509, D510, R511, E513, W535, E536, D539, Y540, and R542^18,25,27,41–43^. The antigen–antibody fragment complex was accurately described using X-ray crystallography. Because viruses propagated with neutralizing antibodies can evolve to escape neutralization, these escape mutants can serve as tools for mapping the epitopes of nAbs. Three MERS-CoV variants generated by KNIH90-F1 antibody treatment had two or three mutations in the RBD region, resulting in the decreased neutralizing activity of KNIH90-F1. Substitutions of residues L506F, R511S, R511L, T512I, T512A, and E513A were identified in the RBD region of these escape mutants. From the neutralizing epitopes previously studied, the substitutions at L506, R511, and E513 were known to inhibit the neutralizing ability of KNIH90-F1. With respect to their ability to escape KNIH90-F1-neutralization, the 4EM (L506F, R511L, and E513A) and 6EM (R511S and T512A) mutants were relatively better than the 1EM mutant (L506F, T512I, and E513A). From this result, we postulated that the substitutions R511L or L in RBD may play a key role in viral escape from KNIH90-F1. The T512 side chain is known to form an important hydrogen bond with D509, although it does not make direct contact with hDPP4; therefore, amino acid substitution at this site may result in decreased hDPP4 binding through the loss of the hydrogen bond formed with D509^36^. Thus, T512I or T512A substitutions at the RBD region of the two escape mutants 1EM and 6EM may contribute to delayed viral growth followed by a decrease in hDPP4 binding due to the lost hydrogen bond between T512 and D509. Analysis of the MERS-CoV mutants that escaped KNIH90-F1 antibody treatment showed that L506, 509, R511, and E513 in the RBD region play critical roles in nAb KNIH90-F1 binding.

**Figure 6:**
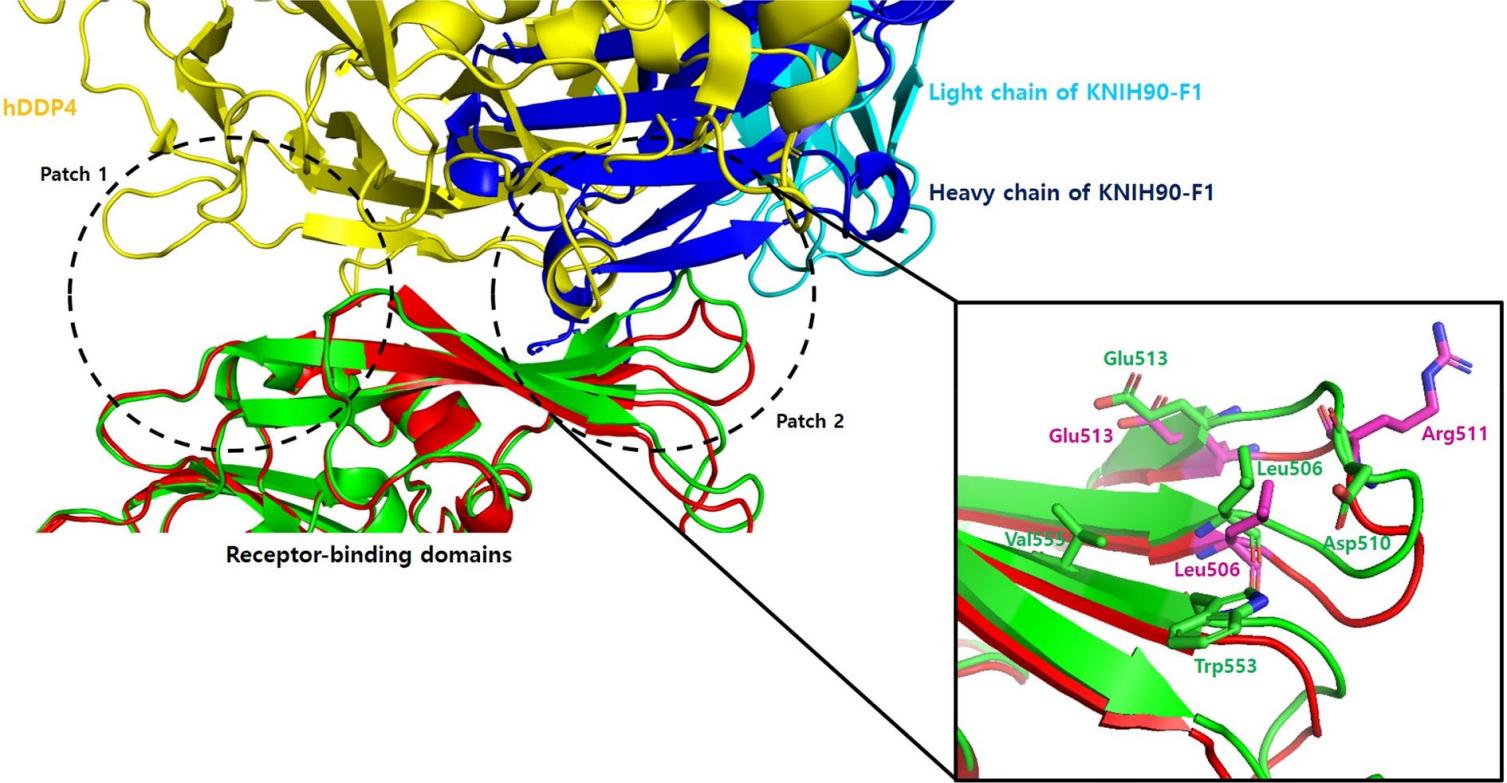
Binding interfaces of MERS-CoV RBD in the complex of hDDP4 (in green) and KNIH90-F1 (in red). hDDP4 interacts with MERS-CoV RBD in patch 1 and 2, while KNIH90-F1 interacts with MERS-CoV RBD in patch 2. Two residues of RBD (L506 and E513) in the binding interface with hDDP4 are overlapped with the epitopes of KNIH90-F1.

The RBD residues of MERS-CoV that interact with hDPP4 were classified into patch 1, which consists of Y499, E536, ED537, and D539, and patch 2, comprising hydrophobic L506, W553, and V555 and hydrophilic D510, R511, and E513^16^. Our results show that the KNIH90-F1 epitopes mainly correspond to patch 2 (Fig. 6). Concurrent with the analysis of the viral mutations in response to KNIH90-F1selective pressure, analysis of the crystal structure of the MERS-CoV complexed with KNIH90-F1 Fab confirmed that L506, R511, and E513 are key residues in the interaction between MERS-CoV and KNIH90-F1 Fab. L506 and E513 of MERS-CoV RBD with DPP4 are overlapped with the epitopes of KNIH90-F1. While hydrophilic R511 of MERS-CoV RBD interacted with hDDP4 and mutation of R511 to Ala did not significantly disrupt the interaction of MERS-CoV RBD with hDDP4, our results show that R511 side chain mainly played a role in the hinge buried by both the heavy and light chains of KNIH90-F1. The positive region in RBD induced by R511 enhances the overall interaction between MERS-CoV RBD and KNIH90-F1 Fab. Thus, the respective substitutions of the positive residue, arginine, with leucine and serine in the escape mutants R511L and R511S inhibited the key interaction between MERS-CoV RBD and KNIH90-F1 Fab by abolishing the hydrogen bond between the amine group in the arginine side chain with the carbonyl group of Tyr115 in the heavy chain and Tyr92 in the light chain of KNIH90-F1. Moreover, the negatively charged residue, E513, in RBD interacts with H114 in the heavy chain of KNIH90-F1 Fab by hydrogen bonding at close proximity, and hydrogen bonding with H114 was inhibited in the escape mutant E513A. L506 is one of the main residues to form the hydrophobic groove in RBD that is involved in the hydrophobic interaction between MERS-CoV and KNIH90-F1 Fab. The substitution of leucine with the relatively congested and bulky residue, phenylalanine, in the escape mutant L506F inhibited the hydrophobic interactions between MERS-CoV RBD and the heavy chain in KNIH90-F1 Fab by steric repulsion.

Consequently, analysis of viral mutations under KNIH90-F1 selective pressure and the crystal structure of MERS-CoV RBD–KNIH90-F1 Fab complex identified the epitope region and the key residues involved in the binding of the complex. Furthermore, our work provides key information for the practical application of KNIH90-F1, along with a cocktail of other nAbs, in targeting various RBD or non-RBD epitopes for MERS treatment and prevention.

## Methods

### Cloning, expression, and purification

The nucleic acid sequence of MERS-CoV RBD (amino acids 367 to 588) of the spike glycoprotein was designed and ordered from IDT. DNA encoding the MERS-CoV RBD with an N-terminal GP67 signal peptide for secretion and a C-terminal 6× His-tag for purification was cloned into the pFasTBac1 vector using the restriction enzyme sites *Eco*RI and *Xho*I. The plasmid was transformed into bacterial DH10Bac competent cells to obtain a bacmid. The bacmid was transfected into Sf9 cells using Cellfectin reagent (Gibco), and the viruses were amplified in four phases. The protein was expressed in Sf9 cells for 72 h at 27 °C. The secreted protein was harvested from the medium and concentrated to 100 ml followed by buffer exchange in HBS buffer containing 10 mM HEPES, pH 7.2, and 150 mM NaCl by overnight dialysis at 4 °C using sartorius vivaflow 200 10 K MWCO PES. The protein was captured with Ni–NTA affinity resin (Qiagen) and washed with 20 mM Imidazole in HBS buffer. The bound protein was eluted with 500 mM Imidazole in HBS buffer, and the eluted protein was further purified using a HiLoad 16/600 Superdex 200 prep grade column (GE Heathcare) with HBS buffer. The purified protein was digested with endoglycosidase F1 and F3 (R&D Systems) overnight at room temperature and purified using Superdex 200 column.

### Generation of KNIH90-F1 Fab fragment and creation of MERS-CoV RBD–KNIH90-F1 Fab complex

**Figure 7:**
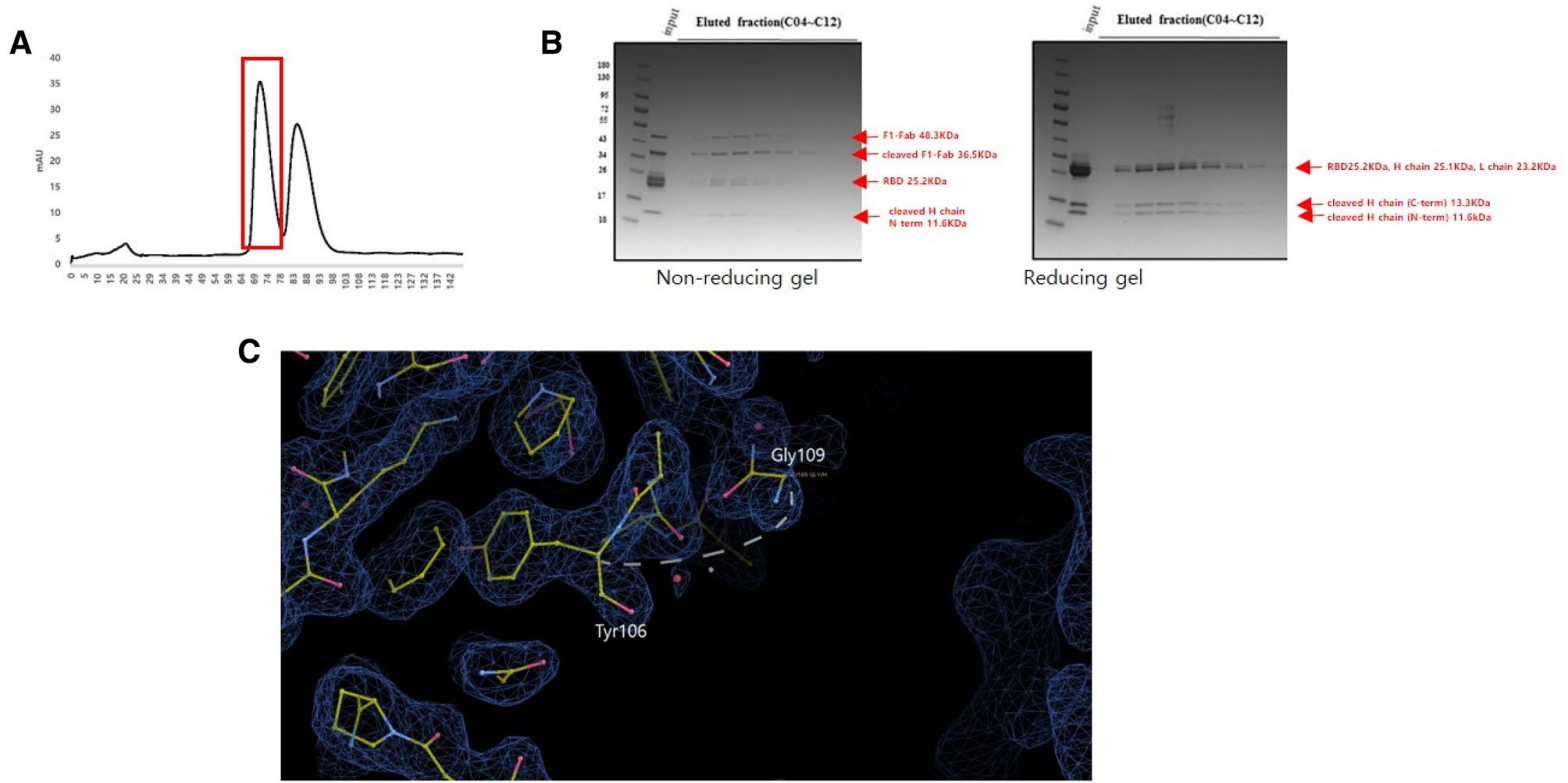
Purification of MERS-CoV RBD in the complex of KNIH90-F1 Fab. (**A**) Size exclusion chromatography. The complex peak is indicated in red. (**B**) SDS-PAGE analysis of eluted faction of RBD and KNIH90-F1 complex. Non-reducing gel (left) and reducing gel (right). (**C**) Cleaved site by papain in heavy chain of KNIH90-F1. G107 and S108 are missing with lack of electron density.

The purified KNIH90-F1 IgG was digested with papain to obtain the KNIH90-F1 Fab fragment. After a 15 min activation incubation, KNIH90-F1 IgG with papain was further incubated for 1 h at 37 °C. Iodoacetamide was added to terminate the digestion. The mixture of Fab and fragment crystallizable (Fc) was separated using a MabSelect column in running buffer containing 20 mM Na_2_HPO_4,_ pH 7.0, and 150 mM NaCl. The Fab fragment was collected from the flow-through and purified using a Superdex 200 column with HBS buffer. The purified MERS-CoV RBD (~ 25 kDa) and KNIH90-F1 Fab (~ 48 kDa) were mixed at a molar ratio of 1:1 and incubated overnight at 4 °C. The complex was purified using Superdex 200 and concentrated to ~ 8 mg/ml in HBS buffer (Fig. 7). The several bands were observed in SDS-PAGE (B in Fig. 7). We found that papain arbitrarily cleaved amino acids between Y106 – G107 and S108 – G109 in heavy chain of KNIH90-F1 Fab, and two residues (G107 and S108) were missing with the lack of electron density in the crystal structure of heavy chain of KNIH90-F1 Fab (C in Fig. 7).

### Crystallization

All crystallization steps were conducted at 18 °C. Any precipitated material of MERS-CoV complexed with KNIH90-F1 Fab was removed by centrifugation prior to the initial screening trials. Initial crystallization trials of the complex using commercial screens were carried out using mosquito liquid handler (TTP Labtech). The ideal crystals were grown in a buffer containing 0.1 M MES monohydrate at pH 6.3 and 15% (w/v) polyethylene glycol 20,000. The size up of crystals was carried out using hanging-drop vapor diffusion in which drops containing 1 μl of protein and 1 μl of mother liquor were equilibrated above a 400-μl mother liquor reservoir. The crystals were soaked in 20% (v/v) ethylene glycol for cryopreservation and quickly frozen in liquid nitrogen on a mount prior to X-ray diffraction.

### Data collection and structural determination

Diffraction data of 2.05 Å resolution were collected at the 5C beamline of the Pohang Accelerator Laboratory, Republic of Korea. Data processing and scaling were performed using the HKL2000^30^. The structure was determined by the molecular replacement phasing method using the CCP4 suite of the program^33^. Model building and refinement were carried out using Coot^34^ and Phenix^35^. Structure validation was performed with the program PROCHECK, and the structure refinement statistics are listed in Table 1. The coordinate and structural factors of the complex structure have been deposited in the Protein Data Bank (PDB ID: 7COE).

### Isolation of antibody escape mutants under selective pressure

We incubated 1 × 10^5^ PFU of the MERS-CoV EMC/2012 strain with 0.1 µg of KNIH90-F1 nAb in 200 µl of medium at 37 °C for 1 h, and then inoculated the virus onto monolayered Vero cells (CCL-81, ATCC, Manassas, VA) for 1 h. After removing the inoculum, and washing three times with PBS, medium containing the same concentration of nAb was added. Development of the cytopathic effect was monitored for 5 days, and progeny viruses were harvested. Then, nAb treatment was repeated for three additional passages. The respective passaged viruses were isolated by plaque purification in the presence of the nAb. The RBD genes of individual isolated viruses were sequenced to identify the mutations that contributed to their escape from the nAb KNIH90-F1.

### Neutralizing activity of escape mutants

The neutralizing assay was conducted using a PRNT assay as described previously with slight modification^36^. Briefly, serial dilutions of nAb were incubated with 80 PFU of mutants isolated by four passages for 1 h at 37 °C. The nAb-mutant mixtures were added to the monolayer of Vero cells and incubated for 1 h. Carboxymethyl cellulose (1.5%) was overlaid, and the plaques formed in each dilution were counted for 3 days following infection. The EMC/2012 strain was used as the virus control.

### Viral growth kinetics

Vero cells incubated in a 6-well plate were infected with the EMC/2012 strain or three escape mutants at a multiplicity of infection of 0.01 for 1 h. After removing the inoculum and washing three times with phosphate-buffered saline (PBS), medium containing 2% fetal bovine serum (FBS) was added. Cell culture supernatants were obtained at various time points and stored at − 70 °C until further use. The viral titers of these supernatants were determined by plaque assay.
